# Forecasting CO_2_ Emissions Using A Novel Grey Bernoulli Model: A Case of Shaanxi Province in China

**DOI:** 10.3390/ijerph19094953

**Published:** 2022-04-19

**Authors:** Huiping Wang, Zhun Zhang

**Affiliations:** Western Collaborative Innovation Research Center for Energy Economy and Regional Development, Xi’an University of Finance and Economics, Xi’an 710100, China; zhunzhang@xaufe.edu.cn

**Keywords:** fractional opposite-direction accumulation, background value, NFOGBM(1,1,*α*,*β*), CO_2_ emissions, forecasting

## Abstract

Accurate predictions of CO_2_ emissions have important practical significance for determining the best measures for reducing CO_2_ emissions and accomplishing the target of reaching a carbon peak. Although some existing models have good modeling accuracy, the improvement of model specifications can provide a more accurate grasp of a system’s future and thus help relevant departments develop more effective targeting measures. Therefore, considering the shortcomings of the existing grey Bernoulli model, in this paper, the traditional model is optimized from the perspectives of the accumulation mode and background value optimization, and the novel grey Bernoulli model NFOGBM(1,1,α,β) is constructed. The effectiveness of the model is verified by using CO_2_ emissions data from seven major industries in Shaanxi Province, China, and future trends are predicted. The conclusions are as follows. First, the new fractional opposite-directional accumulation and optimization methods for background value determination are effective and reasonable, and the prediction performance can be enhanced. Second, the prediction accuracy of the NFOGBM(1,1,α,β) is higher than that of the NGBM(1,1) and FANGBM(1,1). Third, the forecasting results show that under the current conditions, the CO_2_ emissions generated by the production and supply of electricity and heat are expected to increase by 23.8% by 2030, and the CO_2_ emissions of the other six examined industries will decline.

## 1. Introduction

Since the industrial revolution, due to the combustion of fossil fuels such as coal and oil and continuous deforestation, the concentration of CO_2_ in the atmosphere has increased significantly [1]. In recent years, with the continuous and rapid growth of CO_2_ emissions, global warming is a real problem that needs to be solved along with the involvement of scientists. The international community has made active efforts to jointly address climate change, formulate important documents, and provide a basic political framework and legal system for action to address climate change. By the end of 2020, more than 40 countries and economies around the world officially announced carbon neutrality targets. China has the second-largest economy globally, and fossil energy consumption and CO_2_ emissions there continue to grow, accounting for increasing proportions of the global totals. According to the BP Statistical Review of World Energy 2021, China’s CO_2_ emissions in 2020 were 9899.33 million tons, accounting for 30.7% of the global total and ranking first worldwide. Considering the seriousness of climate change, in September 2020, China proposed goals to reach peak CO_2_ emissions by 2030 and achieve carbon neutrality by 2060. At the end of 2021, China successively issued new policies and action plans to meet these carbon peak and carbon neutrality objectives, as well as related medium- and long-term goals. Generally, peak carbon refers to CO_2_ emissions reaching a maximum in a certain year and then entering a declining phase. Carbon neutrality refers to the total CO_2_ produced by a particular organization or society being absorbed and offset over a period of time by natural and artificial means such as afforestation, ocean absorption, engineering storage, etc., to achieve relatively “zero” CO_2_ emissions from human activities.

Shaanxi Province is rich in natural resources and ranks among the top provinces in China in terms of coal and oil production, making it an important energy and chemical industrial base in China, but its CO_2_ emissions have long been high. Under the background of the “double carbon” target, Shaanxi has formulated strict CO_2_ emissions reduction targets. To achieve these goals, it is necessary to understand the current CO_2_ emissions situation in Shaanxi, reasonably predict future CO_2_ emissions and structural characteristics of CO_2_-emitting industries, and formulate reasonable carbon emission reduction measures. Therefore, the rational prediction of CO_2_ emissions has become an important research topic in emissions studies of Shaanxi.

At present, many methods are used to predict CO_2_ emissions, and they can be divided into five categories. The first includes macroeconomic system models. Scholars often analyze and predict CO_2_ emissions based on macroeconomic operational mechanisms, including input–output models, CGE models, the LEAP model, etc. [2,3,4]. The second category includes system optimization models. By comprehensively considering the effects of social and economic development, resource endowment, energy-saving technology, environmental constraints, and consumption behaviour, future CO_2_ emissions can be analyzed and predicted, and corresponding models include the IPAC model and the IAMC model [5,6]. The third category includes index decomposition models. The factors that influence CO_2_ emissions are identified, and the CO_2_ emissions trend is predicted according to the analysis results, and corresponding models include the Kaya, IPAT, and STIRPAT models [7,8,9]. The fourth category includes artificial intelligence models. With the development of information technology, artificial intelligence methods, including artificial neural networks, support vector machines, limit learning machines, etc., have been used for CO_2_ emissions prediction [10,11,12]. The fifth category includes grey prediction models. Most of the above models require including the factors that influence CO_2_ emissions to ensure the effectiveness of the prediction results, but adding variables often increases the time and cost required for data collection, and some data are difficult or even impossible to collect. Grey methods solve this problem and directly model scenarios without considering the effects of other factors, thus requiring limited data to obtain accurate predictions. Therefore, the grey prediction method is widely used for CO_2_ emissions prediction in different regions, industries, and countries because of its convenient operation, simple modeling process, and high accuracy [13,14,15,16]. In addition, grey models have been widely employed in cases involving electric power [17,18], renewable energy [19], natural gas [20], tight gas [21], air pollution [22], traffic flows [23], industrial development [24], landslides [25], and COVID-19 [26,27].

Grey prediction modeling is the most active and widely explored branch of grey system theory, and it is also a new research direction in mainstream prediction theory [28]. However, the core model GM(1,1) has some shortcomings, such as limited adaptability and unstable performance; therefore, scholars have improved this model from many perspectives. For example, the accumulation method was optimized by adding opposite-direction accumulation, fractional accumulation, and comfortable accumulation to improve modeling performance [29,30,31]. Additionally, the continuity of data sequences was improved by processing the original data [32,33], and the modeling error was reduced by improving the initial values of the model [34]. Moreover, the calculation of background values, such as through deriving background values based on a nonhomogeneous exponential function, has been improved [35], and Simpson’s law was used to optimize background values [36]. To improve the prediction performance of the model, the quantum optimization [37], grey wolf optimization (GWO) [38], particle swarm optimization (PSO) [39], and many other intelligent optimization algorithms have been applied to find the optimal parameters. The structure of a grey model can also be optimized, such as by changing the grey processes in the model and replacing them with binomial, polynomial, or other functions to build a new model [40,41]. As new information is more helpful than old information in trend assessment, prioritizing new information has become a common way to improve the prediction accuracy of grey models. Scholars often use a rolling prediction mechanism with metabolic thought to establish optimization models; this approach can not only prioritize new information but also prolong the prediction period of results [42,43,44]. The rolling prediction mechanism ensures that the modeling data are up to date by continuously adding new prediction data, which effectively improves the modeling result. In addition, the GM(1,1) model can be combined with artificial intelligence and statistical analysis models to improve the overall prediction accuracy [45,46,47].

However, most of the above models are linear, and there are many nonlinear problems in most real-world scenarios. Chen et al. [48] first proposed a new nonlinear Bernoulli model NGBM(1,1) to predict the exchange rates of Taiwan’s main trading partners and achieved good prediction results. The nonlinear grey Bernoulli model, which is also known as the GM(1,1) power model, can produce results that highly fit the cumulative curve of the original sequence by adjusting the weight index η, and it has displayed good prediction performance for nonlinear problems. Moreover, when η = 2, this model is called the grey Verhulst model, which is ideal for data that plot along an S-shaped curve. Based on the effectiveness of the grey Bernoulli model in yielding accurate predictions for nonlinear problems, many scholars have optimized NGBM(1,1) from different perspectives. For example, Guo et al. [49] combined the principle of self-memory with an optimized nonlinear grey Bernoulli model to overcome a major weakness of the traditional Bernoulli model—its sensitivity to the initial parameter values. Wu et al. [19] applied fractional accumulation to the grey Bernoulli model to construct the FANGBM(1,1) model and predicted renewable energy consumption in China. Şahin [50] introduced seasonal factors based on the FANGBM(1,1) model, proposed the OFANGBM(1,1) model, and predicted power generation and installed capacities in Turkey. Xie et al. [15] adopted a simple form of conformable fractional calculations and established the CCFNGBM(1,1) model to predict CO_2_ emissions related to fuel combustion. Zheng et al. [20] proposed the CFNHGBM(1,1,K) model based on the moth-flame optimization (MFO) algorithm to predict natural gas production and consumption. Wang and Wang [51] combined the NGBM(1,1) and FPGM(1,1,$t^{\alpha }$) models and proposed GFBGM(1,1,$t^{\alpha }$), which was used to predict the per capita primary energy consumption of major economies around the world.

Each improvement of the model specifications contributes to a more accurate prediction of the examined system’s future, thus providing more effective reference values for relevant sectors. Therefore, based on the above research and considering the unique background value error of the traditional grey Bernoulli model, a new background value optimization method is proposed in this paper. Moreover, based on a new fractional opposite-directional accumulation operation, a novel grey Bernoulli model NFOGBM(1,1,α,β) is established. Based on the carbon emission data obtained from the Shaanxi Provincial Statistical Yearbook, it is found that carbon emissions in Shaanxi Province occur mainly in seven major industries: wholesale, retail trade and catering services (WRTCS), petroleum and natural gas extraction (PNGE) transportation, storage, postal and telecommunication services (TSPTS), smelting and pressing of ferrous metals (SPFM), nonmetal minerals mining and dressing (NMMD), coal mining and dressing (CMD), and the production and supply of electric power, steam, and hot water (PSESH). Therefore, this paper uses these seven data sets to verify the validity of the NFOGBM(1,1,α,β) model, and the CO_2_ emissions from seven industries from 2020 to 2030 in Shaanxi Province, China, are predicted by using the new model and a metabolic concept. The main contributions are summarized as follows:

(1) Considering the unique background value error in the nonlinear grey Bernoulli model, a new optimization method is proposed to further reduce the background value error and improve the prediction performance of the model.
(2) By combining the new fractional opposite-direction accumulation operation, background value optimization, and the FANGBM(1,1) model, the NFOGBM(1,1,α,β) model is constructed, and its optimal parameters are determined with the PSO algorithm.
(3) The effectiveness of the NFOGBM(1,1,α,β) model is verified by using CO_2_ emissions data from seven major industries in Shaanxi, China. The results show that the model outperforms other methods. Notably, unlike the NGBM(1,1) model, it avoids the insufficient utilization of new information, and overfitting, which limits the FGNOM(1,1) model, is also avoided.
(4) Based on a metabolic concept, the NFOGBM(1,1,α,β) model is used to predict the CO_2_ emissions of seven major industries in Shaanxi Province.

The rest of this paper is organized as follows. In Section 2, a new fractional-order opposite-direction accumulation grey Bernoulli model NFOGBM(1,1,α,β) is proposed. In Section 3, the effectiveness of the NFOGBM(1,1,α,β) model is verified by using CO_2_ emissions data from seven major industries in Shaanxi Province, China, and CO_2_ emissions from 2020 to 2030 are forecasted based on a metabolic concept. The conclusions of the study are given in Section 4.

## 2. New Fractional Opposite-Direction Accumulation Grey Bernoulli Model NFOGBM(1,1,α,β)

In this section, the traditional nonlinear grey Bernoulli model NGBM(1,1) [48] and fractional nonlinear grey Bernoulli model FANGBM(1,1) [19] are briefly introduced, and then, a new fractional opposite-direction accumulation operation and a background value optimization method are proposed. Finally, a new fractional opposite-direction accumulation grey Bernoulli model NFOGBM(1,1,α,β) is constructed.

### 2.1. NGBM(1,1) and FANGBM(1,1) Models

**Definition** **1.** *Consider a nonnegative sequence* $X^{\left(0\right)}={\left(x^{\left(0\right)}(1),\;x^{\left(0\right)}(2),\;x^{\left(0\right)}(3)\ldots x^{\left(0\right)}(n)\right)}^{T}$*, where* T *represents the transpose operation*. $X^{\left(r\right)}=D^{r}X^{\left(0\right)}$ *is called the* r*-order accumulation sequence of the original sequence, and* $D^{r}$ *is the* r*-order accumulation generation matrix,* $X^{\left(r\right)}={\left(x^{\left(r\right)}(1),\;x^{\left(r\right)}(2),\;x^{\left(r\right)}(3)\ldots x^{\left(r\right)}(n)\right)}^{T}$*, where*

(1) $$x^{\left(r\right)}\left(k\right)=d^{r}x^{\left(0\right)}\left(k\right)=\overset{k}{\underset{i=1}{\sum }}\frac{\Gamma \left(r+k-i\right)}{\Gamma \left(k-i+1\right)\Gamma \left(r\right)}x^{\left(0\right)}\left(i\right),\;k=1,2\ldots ,n\;$$

(2) $$D^{r}={\left[\begin{array}{ccccc} \left[\begin{array}{c} r\\ 0 \end{array}\right] & 0 & 0 & \cdots & 0\\ \left[\begin{array}{c} r\\ 1 \end{array}\right] & \left[\begin{array}{c} r\\ 0 \end{array}\right] & 0 & \cdots & 0\\ \left[\begin{array}{c} r\\ 2 \end{array}\right] & \left[\begin{array}{c} r\\ 1 \end{array}\right] & \left[\begin{array}{c} r\\ 0 \end{array}\right] & \cdots & 0\\ \vdots & \vdots & \vdots & \ddots & 0\\ \left[\begin{array}{c} r\\ n-1 \end{array}\right] & \left[\begin{array}{c} r\\ n-2 \end{array}\right] & \left[\begin{array}{c} r\\ n-3 \end{array}\right] & \cdots & \left[\begin{array}{c} r\\ 0 \end{array}\right] \end{array}\right]}_{n\times n}$$

*where* $\left[\begin{array}{c} r\\ i \end{array}\right]=\{\begin{array}{c} \frac{r\left(r+1\right)\cdots \left(r+i-1\right)}{i!}=\left(\begin{array}{c} r+i-1\\ i \end{array}\right)=\frac{\left(r+i-1\right)!}{i!\left(r-1\right)!},r\in Z\\ \frac{\Gamma \left(r+i\right)}{\Gamma \left(i+1\right)\Gamma \left(r\right)},\alpha \notin Z \end{array},\left[\begin{array}{c} 0\\ i \end{array}\right]=0,\left[\begin{array}{c} 0\\ 0 \end{array}\right]=1$*,*$d^{r}$*is calledr-order accumulation generation operator (r-AGO), and*$Z^{\left(r\right)}={\left(z^{\left(r\right)}(1),\;z^{\left(r\right)}(2),\;z^{\left(r\right)}(3)\ldots z^{\left(r\right)}(n)\right)}^{T}$*is the background value sequence of the original series, where*

(3) $$z^{\left(r\right)}(k)=0.5x^{\left(r\right)}(k-1)+0.5x^{\left(r\right)}(k)\;$$

*Then*

(4) $$\frac{dx^{\left(r\right)}}{dt}+ax^{\left(r\right)}=b{\left(x^{\left(r\right)}\right)}^{\eta }\;$$

*is called the fractional-order nonlinear grey Bernoulli model FANGBM(1,1), where* η*is any real number. When*r=1*, this model is the nonlinear grey Bernoulli model NGBM(1,1). If both sides of Equation (4) are integrated into the interval [k − 1, k], Equation (4) can be rewritten as*

(5) $$x^{\left(r\right)}(k)-x^{\left(r\right)}(k-1)+a\int_{k-1}^{k}x^{\left(r\right)}(t)dt=b\int_{k-1}^{k}{\left(x^{\left(r\right)}(t)\right)}^{\eta }dt\;$$

*where* $\int_{k-1}^{k}x^{\left(r\right)}(t)dt$*and*$\int_{k-1}^{k}{\left(x^{\left(r\right)}(t)\right)}^{\eta }dt$*are the areas enclosed by the functions*$x^{\left(r\right)}(t)$*and*${\left(x^{\left(r\right)}(t)\right)}^{\eta }$*, respectively, and the*t*axis in the interval [k −1, k]; thus, the corresponding variables can be approximately set as* $z^{\left(r\right)}(k)$*and*${\left(z^{\left(r\right)}(k)\right)}^{\eta }$*, and Equation (5) can be rewritten as*

(6) $$x^{\left(r\right)}(k)-x^{\left(r\right)}(k-1)+az^{\left(r\right)}(k)=b{\left(z^{\left(r\right)}(k)\right)}^{\eta }$$

*Therefore, according to the least squares method, the parameters in the model can be calculated based on the following formula:*

(7) $${\left(\begin{array}{cc} \hat{a}, & \hat{b} \end{array}\right)}^{T}={\left(B^{T}B\right)}^{-1}B^{T}Y\;$$

*where*

(8) $$B=\left[\begin{array}{cc} -z^{\left(r\right)}(2) & {\left(z^{\left(r\right)}(2)\right)}^{\eta }\\ -z^{\left(r\right)}(3) & {\left(z^{\left(r\right)}(3)\right)}^{\eta }\\ \vdots & \vdots \\ -z^{\left(r\right)}(n) & {\left(z^{\left(r\right)}(n)\right)}^{\eta } \end{array}\right],\;Y=\left[\begin{array}{c} x^{\left(r\right)}(2)-x^{\left(r\right)}(1)\\ x^{\left(r\right)}(3)-x^{\left(r\right)}(2)\\ \vdots \\ x^{\left(r\right)}(n)-x^{\left(r\right)}(n-1) \end{array}\right]\;$$

*By solving Equation (4), the corresponding function of the final time of the model can be* *obtained:*

(9) x^(r)(k)=[((x(r)(1))1−η−b/a)e−a(1−η)(k−1)+b/a]11−η,k=1,2…,n 

*The final reduction value can be calculated according to the fractional subtraction generation operator (* *r-IAGO):*

(10) $${\hat{x}}^{\left(0\right)}\left(k\right)=d^{-r}{\hat{x}}^{\left(r\right)}\left(k\right)=\overset{k-1}{\underset{i=0}{\sum }}{\left(-1\right)}^{i}\frac{\Gamma \left(r+1\right)}{\Gamma \left(i+1\right)\Gamma \left(r-i+1\right)}{\hat{x}}^{\left(r\right)}\left(k-i\right),\;k=1,2\ldots ,n\;$$

### 2.2. New Fractional Opposite-Direction Accumulation Operation

**Definition** **2.** *Consider a nonnegative sequence* $X^{\left(0\right)}={\left(x^{\left(0\right)}(1),\;x^{\left(0\right)}(2),\;x^{\left(0\right)}(3)\ldots x^{\left(0\right)}(n)\right)}^{T}$*, where* T represents the transpose operation. $X^{\left(r\right)}=N^{r}X^{\left(0\right)}$ is called the new r-order opposite-direction accumulation sequence of the original sequence, and $N^{r}$ *is the r-order new opposite-direction accumulation generation matrix,* $X^{\left(r\right)}={\left(x^{\left(r\right)}(1),\;x^{\left(r\right)}(2),\;x^{\left(r\right)}(3)\ldots x^{\left(r\right)}(n)\right)}^{T}$*, where*

(11) $$x^{\left(r\right)}\left(k\right)=n^{r}x^{\left(0\right)}\left(k\right)==\overset{k}{\underset{i=1}{\sum }}\frac{\Gamma \left(r+i-1\right)}{\Gamma \left(i\right)\Gamma \left(r\right)}x^{\left(0\right)}\left(i\right),\;k=1,2\ldots ,n\;$$

*where* $n^{r}$ *is called the r-order accumulation generation operator (r-NOAGO). Matrix* $N^{r}$ *can be expressed as*

(12) $$N^{r}={\left[\begin{array}{ccccc} \left[\begin{array}{c} r\\ 0 \end{array}\right] & 0 & 0 & \cdots & 0\\ \left[\begin{array}{c} r\\ 0 \end{array}\right] & \left[\begin{array}{c} r\\ 1 \end{array}\right] & 0 & \cdots & 0\\ \left[\begin{array}{c} r\\ 0 \end{array}\right] & \left[\begin{array}{c} r\\ 1 \end{array}\right] & \left[\begin{array}{c} r\\ 2 \end{array}\right] & \cdots & 0\\ \vdots & \vdots & \vdots & \ddots & 0\\ \left[\begin{array}{c} r\\ 0 \end{array}\right] & \left[\begin{array}{c} r\\ 1 \end{array}\right] & \left[\begin{array}{c} r\\ 2 \end{array}\right] & \cdots & \left[\begin{array}{c} r\\ n-1 \end{array}\right] \end{array}\right]}_{n\times n}$$

*As shown above, matrix* $N^{r}$ *and matrix* $D^{r}$ *satisfy* $D^{r}{}_{i,j}=N^{r}{}_{i,i-j+1},\;i,j<n$*. The new opposite-direction subtraction operator (*r*-INOAGO) corresponding to the new opposite-direction accumulation operator is*

(13) $$x^{\left(0\right)}\left(k\right)=n^{-r}x^{\left(r\right)}\left(k\right)=\frac{\Gamma \left(k\right)\Gamma \left(r\right)}{\Gamma \left(r+k-1\right)}(x^{\left(r\right)}\left(k\right)-x^{\left(r\right)}\left(k-1\right)),k=2,3\ldots ,n$$

New information prioritization is an important principle in grey system modeling; that is, the newest data in the original sequence provide the most valuable grey information, and accordingly, compared with old data, we should prioritize new data in the modeling process. The opposite-direction accumulation operation can be used to prioritize new information [29]. Notably, the extrapolation performance of the traditional model is generally poor, and consequently, the prediction accuracy is low. The new opposite-direction accumulation approach solves this problem by fully considering all available information, thus providing high accuracy and practicality.

### 2.3. Optimization of Background Value

A certain inherent error is associated with the derivation from Equation (5) to Equation (6); that is, functions $\int_{k-1}^{k}x^{\left(r\right)}(t)dt$ and $\int_{k-1}^{k}{\left(x^{\left(r\right)}(t)\right)}^{\eta }dt$ are approximately regarded as $z^{\left(r\right)}(k)$ and ${\left(z^{\left(r\right)}(k)\right)}^{\eta }$, which is unreasonable. Zhou et al. [52] used the following formula to calculate the background value: $\alpha x^{\left(1\right)}(k)+(1-\alpha )x^{\left(1\right)}(k-1)$. This method of setting the background value can reduce the corresponding model error to a large extent. Therefore, in this paper, the background values of the grey Bernoulli model are defined in a related way, as follows.

**Definition** **3.** *Let* $\alpha ,\beta \in \left[\begin{array}{cc} 0, & 1 \end{array}\right]$*, the exact value of which has yet to be determined. The functions* $\int_{k-1}^{k}x^{\left(r\right)}(t)dt$ *and* $\int_{k-1}^{k}{\left(x^{\left(r\right)}(t)\right)}^{\eta }dt$ *are approximated as* $z^{\left(r\right)}(k)$ *and* ${\left(m^{\left(r\right)}(k)\right)}^{\eta }$*, respectively, where*

(14) $$\begin{array}{c} z^{\left(r\right)}(k)=\alpha x^{\left(r\right)}(k-1)+(1-\alpha )x^{\left(r\right)}(k)\\ m^{\left(r\right)}(k)=\beta x^{\left(r\right)}(k-1)+(1-\beta )x^{\left(r\right)}(k)\\ k=2,3,\cdots ,n \end{array}$$

### 2.4. A New Fractional Opposite-Direction Accumulation Grey Bernoulli Model (NFOGBM(1,1,α,β))

The novel fractional opposite-direction accumulation operation, optimization approach for background values, and FANGBM(1,1) model are combined to form the novel fractional opposite-direction accumulation grey Bernoulli model NFOGBM(1,1,α,β), which is defined as follows.

**Definition** **4.** *Consider a nonnegative sequence* $X^{\left(0\right)}={\left(x^{\left(0\right)}(1),\;x^{\left(0\right)}(2),\;x^{\left(0\right)}(3)\ldots x^{\left(0\right)}(n)\right)}^{T}$*, where *T *represents the transpose operation. Then,* $\frac{dx^{\left(r\right)}}{dt}+ax^{\left(r\right)}=b{\left(x^{\left(r\right)}\right)}^{\eta }$ *in NFOGBM(1,1,*α,β*) is used, where* $x^{\left(r\right)}(k)$ *is as shown in Equation (11). By integrating both sides of the equation, we can approximate the resulting expression as*

(15) $$x^{\left(r\right)}(k)-x^{\left(r\right)}(k-1)+az^{\left(r\right)}(k)=b{\left(m^{\left(r\right)}(k)\right)}^{\eta }\;$$

*where* $z^{\left(r\right)}(k)$ *and* $m^{\left(r\right)}(k)$ *are shown in Equation (14). The development coefficient* a *and grey action* b *are given by* ${\left(\begin{array}{cc} \hat{a}, & \hat{b} \end{array}\right)}^{T}={\left(B^{T}B\right)}^{-1}B^{T}Y$ *, according to the least-squares method, where*

(16) $$B=\left[\begin{array}{cc} -z^{\left(r\right)}(2) & {\left(m^{\left(r\right)}(2)\right)}^{\eta }\\ -z^{\left(r\right)}(3) & {\left(m^{\left(r\right)}(3)\right)}^{\eta }\\ \vdots & \vdots \\ -z^{\left(r\right)}(n) & {\left(m^{\left(r\right)}(n)\right)}^{\eta } \end{array}\right],\;Y=\left[\begin{array}{c} x^{\left(r\right)}(2)-x^{\left(r\right)}(1)\\ x^{\left(r\right)}(3)-x^{\left(r\right)}(2)\\ \vdots \\ x^{\left(r\right)}(n)-x^{\left(r\right)}(n-1) \end{array}\right]$$

*The time response equation of the model is* x^(r)(k)=[((x(r)(1))1−η−b/a)e−a(1−η)(k−1)+b/a]11−η*, and the final reduced equation* ${\hat{x}}^{\left(0\right)}(k)$ *is calculated according to Equation (13).*

Based on the definition above, the relationship between the NFOGBM(1,1,α,β) model and other existing grey models can be assessed.

When α=β=0.5, r=1, and η=0, the model becomes the traditional GM(1,1) model [28].

(17) $$\frac{dx^{\left(1\right)}}{dt}+ax^{\left(1\right)}=b$$

When α=β=0.5, *r* = 1, and η=2, the model becomes the grey Verhulst model [21].

(18) $$\frac{dx^{\left(1\right)}}{dt}+ax^{\left(1\right)}=b{\left(x^{\left(1\right)}\right)}^{2}$$

When α=β=0.5, r=1, and η=2, the model becomes the NGBM(1,1) model [48].

(19) $$\frac{dx^{\left(1\right)}}{dt}+ax^{\left(1\right)}=b{\left(x^{\left(1\right)}\right)}^{\eta }$$

When α=β=0.5 and $x^{\left(r\right)}\left(k\right)$ is determined as shown in Equation (1), the model becomes the FANGBM(1,1) model [19].

(20) $$\frac{dx^{\left(r\right)}}{dt}+ax^{\left(r\right)}=b{\left(x^{\left(r\right)}\right)}^{\eta }$$

**Figure 1:**
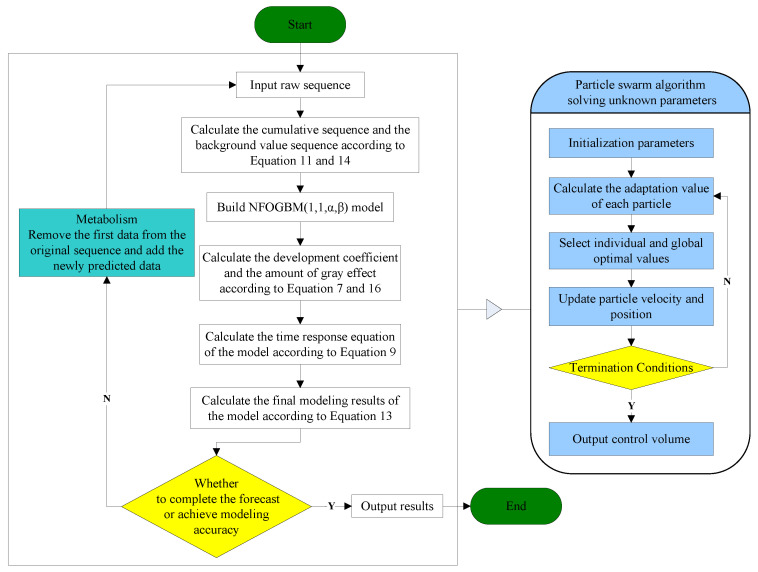
NFOGBM(1,1,α,β) modeling process.

According to Definition 4, the NFOGBM(1,1,α,β) model has a total of six unknown parameters, among which the development coefficient a and grey action b are known, but determining the optimal values of the parameters r, η, α, β remains a challenge. Therefore, in this paper, the PSO algorithm is used to set these four unknown parameters and minimize the mean relative percent error of simulations (MRSPE). The specific calculation steps are shown in Figure 1 with the following equations.

(21) $$\min\mathrm{MRSPE}=\frac{1}{n-1}\sum_{i=2}^{n}\left({\hat{x}}^{\left(0\right)}(i)-x^{\left(0\right)}(i))/x^{\left(0\right)}(i\right)\times 100\%$$

(22) s.t.(z(r)(k)=αx(r)(k−1)+(1−α)x(r)(k)m(r)(k)=βx(r)(k−1)+(1−β)x(r)(k)x(r)(k)=nrx(0)(k)==∑i=1kΓ(r+i−1)Γ(i)Γ(r)x(0)(i)(a^,b^)T=(BTB)−1BTYx^(r)(k)=[((x(r)(1))1−η−b/a)e−a(1−η)(k−1)+b/a]11−ηx^(0)(k)=n−rx(r)(k)=Γ(k)Γ(r)Γ(r+k−1)(x^(r)(k)−x^(r)(k−1))k=2,3⋯,n

### 2.5. Metabolic Ideas and the NFOGBM(1,1,α,β) Modeling Process

When a grey model is used, the prediction accuracy is often less than ideal when the original data are applied for long-term prediction. Notably, over time, the grey factors that influence the considered variables will continue changing, as will the most recent state of the system. If the original data are used to build the grey prediction model, the prediction accuracy of the model will inevitably decrease, and its reliability will also decrease. Therefore, a grey model can generally only obtain good results when predicting one or two values, and the long-term prediction effect is not satisfactory and can only reflect approximate trends. To remedy this deficiency, a metabolic mechanism needs to be used for modeling.

The so-called metabolic concept involves removing the old data from the original modeling sequence and performing the modeling steps again according to the most recent prediction data generated by the model, i.e., after predicting one or two values, the newly predicted values are added to the original sequence, and the old data in the original genus sequence are removed, thus keeping the dimension of the modeling sequence unchanged. Through such a metabolic concept, new prediction information is continuously added, and the grey level can be gradually reduced until the prediction objectives are met or a certain accuracy requirement is reached.

To clearly demonstrate how the NFOGBM(1,1,α,β) model proposed in this paper can be used to solve a practical prediction problem, a flowchart is presented in Figure 1.

### 2.6. Error Metrics

To better verify the reliability and fit of the NFOGBM(1,1,α,β) model, the mean absolute percentage error (MAPE) is used to assess the accuracy of the model. Given that the test of grey model performance includes simulation performance and prediction performance, the original data need to be divided into modeling and prediction subsets. Each index is then evaluated from simulation, prediction, and overall perspectives. The MAPE of the simulation stage is also called the mean relative percentage error of simulations (MRSPE); the MAPE of the prediction stage is also called the mean prediction percentage error (MRFPE), and the overall MAPE is also known as the combined mean relative percentage error (CMRPE). The MRSPE, MRFPE, and CMRPE are calculated as follows:

(23) $$\mathrm{MRSPE}=\frac{1}{n}\overset{n}{\underset{k=1}{\sum }}\left|\frac{x^{\left(0\right)}\left(k\right)-{\hat{x}}^{\left(0\right)}\left(k\right)}{x^{\left(0\right)}\left(k\right)}\right|\times 100\%$$

(24) $$\mathrm{MRFPE}=\frac{1}{t}\overset{n+t}{\underset{k=n+1}{\sum }}\left|\frac{x^{\left(0\right)}\left(k\right)-{\hat{x}}^{\left(0\right)}\left(k\right)}{x^{\left(0\right)}\left(k\right)}\right|\times 100\%$$

(25) $$\mathrm{CMRPE}=\frac{1}{n+t}\overset{n+t}{\underset{k=1}{\sum }}\left|\frac{x^{\left(0\right)}\left(k\right)-{\hat{x}}^{\left(0\right)}\left(k\right)}{x^{\left(0\right)}\left(k\right)}\right|\times 100\%$$

where n is the number of modeling samples and t is the prediction interval.

## 3. Applications in Forecasting Shaanxi’s CO_2_ Emissions

In this paper, the data from the seven industries with the highest CO_2_ emissions in Shaanxi Province are used to verify the prediction performance of the NFOGBM(1,1,α,β) model, and compare it with the NGBM(1,1) model and FANGBM(1,1) model. On this basis, the CO_2_ emissions of the seven industries from 2020 to 2030 are predicted to provide a reference for use by relevant departments in Shaanxi Province when formulating carbon reduction policies. The seven industries are WRTCS, PNGE, TSPTS, SPFM, NMMD, CMD, and PSESH. For convenience, the above three models are abbreviated as NFOGBM, FANGBM, and NGBM, respectively. The PSO algorithm is applied to calculate the unknown parameters, and the settings are as follows: learning factors c1 = c2 = 2, the inertia factor is 0.8, the population size is 50, the maximum number of iterations is 300, the range of the cumulative order is [0, 3], and the range of values is [−2, 5].

### 3.1. Data Description

**Figure 2:**
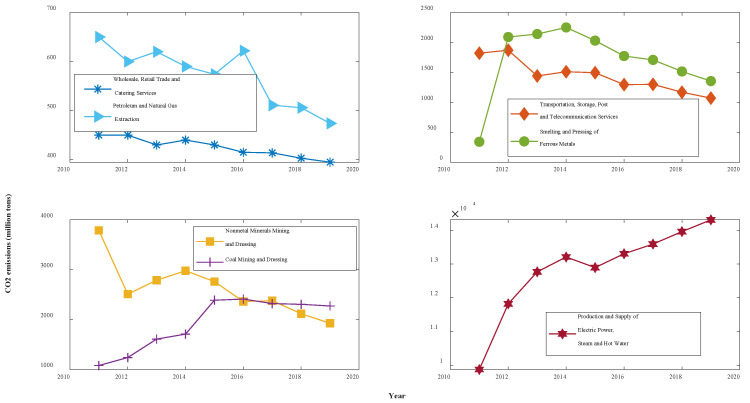
Trends of CO_2_ emissions for 7 industries in Shaanxi, 2011–2019.

In this paper, the carbon emission coefficient method is used to measure the CO_2_ emissions from seven industries in Shaanxi Province, China. The required energy consumption data were obtained from the Shaanxi Provincial Statistical Yearbook, and the carbon emission coefficients and converted standard coal coefficients of various types of energy were based on the IPCC Guidelines for National Greenhouse Gas Inventory and the China Energy Statistical Yearbook. The carbon emissions data for each industry were obtained via calculations, as shown in Table 1 and Figure 2. Among them, WRTCS has the lowest CO_2_ emissions, with an average of only 4.25 million tons over the past nine years, and PSESH yields the highest CO_2_ emissions, with its average of 128.6 million tons totalling more than those of the other six sectors combined. In terms of trend, CO_2_ emissions from four industries, namely, WRTCS, PNGE, TSPTS, and NMMD, have consistently decreased, and CO_2_ emissions from SPFM have been steadily decreasing since 2014. Additionally, CO_2_ emissions from CMD have only slightly decreased since 2016. In contrast, the amount of CO_2_ emitted from PSESH has been increasing at an average annual rate of 4.9%.

### 3.2. Model Comparison

Carbon emissions data from seven industries are analyzed and compared using three models, NFOGBM, FANGBM, and NGBM, to predict data for two years, 2018 and 2019. Then, the simulation, prediction, and overall performance of the three models are assessed.

**Figure 3:**
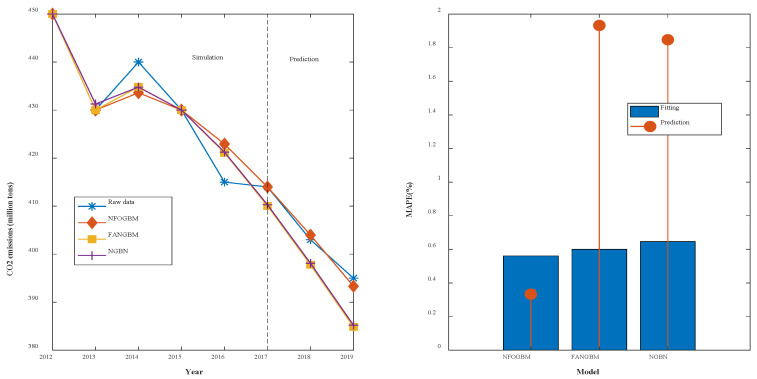
Fitting curves and error plots of the WRTCS results.

**Figure 4:**
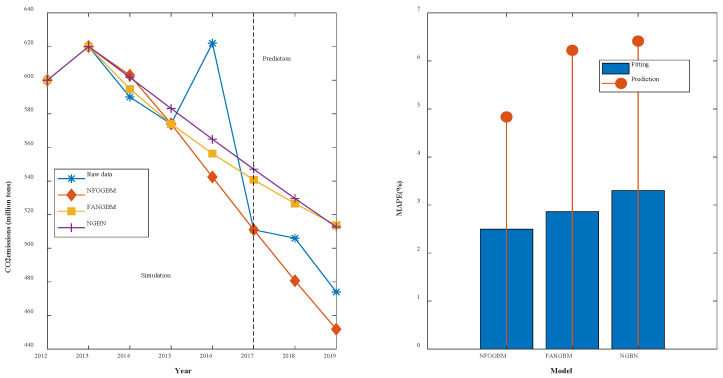
Fitting curves and error plots of the PNGE results.

**Figure 5:**
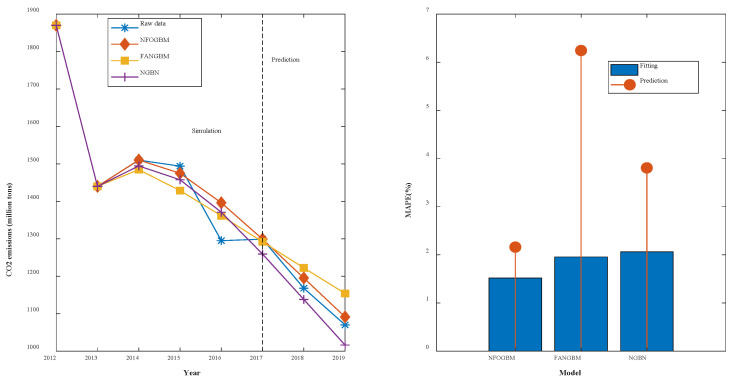
Fitting curves and error plots of the TSPTS results.

**Figure 6:**
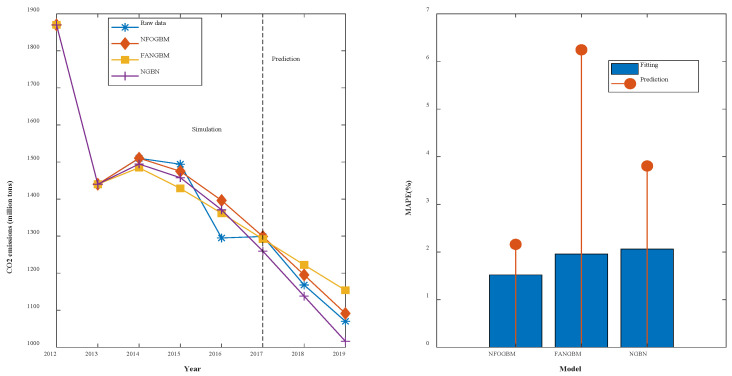
Fitting curves and error plots of the SPFM results.

**Figure 7:**
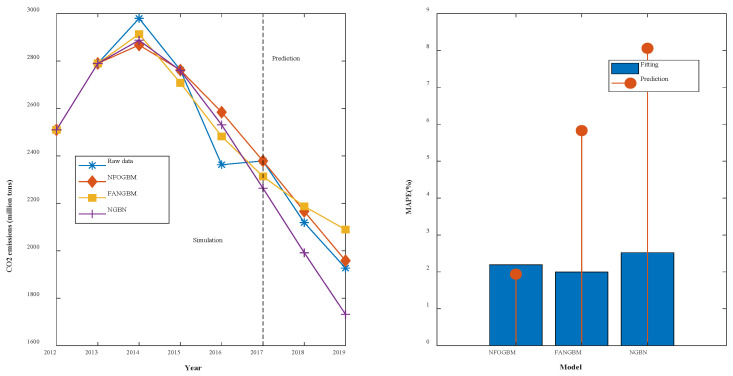
Fitting curves and error plots of the NMMD results.

**Figure 8:**
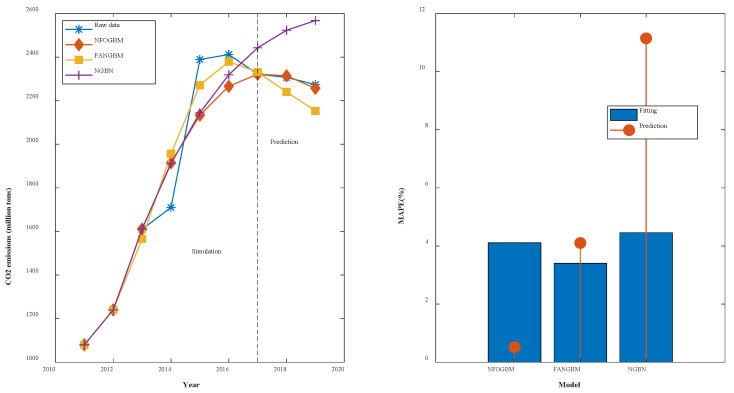
Fitting curves and error plots of the CMD results.

**Figure 9:**
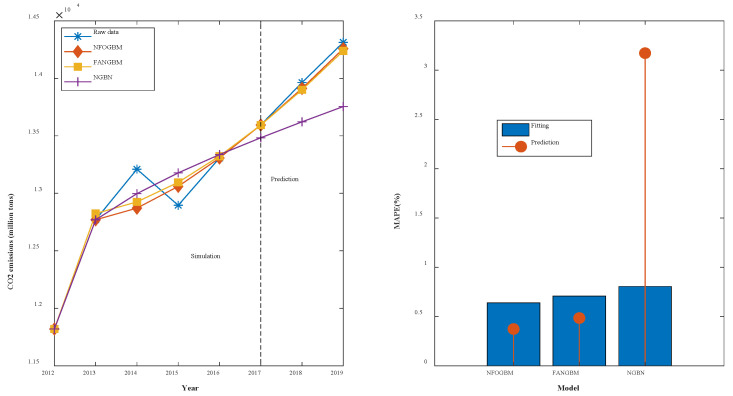
Fitting curves and error plots of the PSESH results.

(1) The modeling results for WRTCS are shown in Table 2, and the fitted curves and error plots are shown in Figure 3. The MRPESs (%) of the NFOGBM, FANGBM, and NGBM are 0.561, 0.6, and 0.647, respectively; the MRFPEs (%) are 0.333, 1.932, and 1.847, respectively, and the CMRPEs (%) are 0.504, 0.933, and 0.947, respectively. It can be seen that the simulation errors of the three models are not very different, but the NFOGBM has a much lower prediction error and integrated error than those of the other two models, indicating that the NFOGBM provides the optimal simulation, prediction, and integrated performance. In addition, the curves obtained with the NFOGBM are closest to the original data, indicating that the model provides the best predictions of future scenarios. It is worth noting that the FANGBM reduces the simulation error to some extent but increases the prediction error compared to the NGBM.

(2) The modeling results for PNGE are shown in Table 3, and the fitting and error plots are shown in Figure 4. The MRPESs (%) of the NFOGBM, FANGBM, and NGBM are 2.498, 2.864, and 3.302, respectively; the MRFPEs (%) are 4.836, 6.222, and 6.415, respectively, and the CMRPEs (%) are 3.084, 3.704, and 4.08, respectively. As in the previous example, the NFOGBM yields the smallest simulation, prediction, and combined errors, and can best capture the trends of the data to produce accurate predictions. It is worth mentioning that the optimal solution of parameter α produced by the NFOGBM in this example is 0, implying that $z^{\left(r\right)}(k)=x^{\left(r\right)}(k)$.

(3) The modeling results for TSPTS are shown in Table 4, and the fitting and error plots are shown in Figure 5. The MRPESs (%) of the NFOGBM, FANGBM, and NGBM are 1.52, 1.957, and 2.062, respectively; the MRFPEs (%) are 2.16, 6.244, and 3.806, respectively, and the CMRPEs (%) are 1.68, 3.028, and 2.498, respectively. In this example, the NFOGBM again yields the smallest simulation, prediction, and synthesis errors, and the highest modeling accuracy. Compared to the NGBM, the FANGBM yields some overfitting, which improves the simulation accuracy but leads to low prediction accuracy. Notably, the optimal solution of parameter α produced by the NFOGBM in this example is 1, implying that $z^{\left(r\right)}(k)=x^{\left(r\right)}(k-1)$.

(4) The modeling results for SPFM are shown in Table 5, and the fitting and error diagrams are shown in Figure 6. The MRPESs (%) of the NFOGBM, FANGBM, and NGBM are 1.759, 1.272, and 2.118, respectively; the MRFPEs (%) are 1.837, 5.619, and 3.231, respectively, and the CMRPEs (%) are 1.779, 2.359, and 2.397. In this example, the FANGBM produces the lowest simulation error but has the highest prediction error, resulting in some overfitting, which can be seen in the fitted graphs. The NFOGBM solves this problem, and yields the lowest prediction and synthesis errors as well as the highest modeling accuracy in this example.

(5) The modeling results for NMMD are shown in Table 6, and the fitting and error plots are shown in Figure 7. The MRPESs (%) of the NFOGBM, FANGBM, and NGBM are 2.191, 1.998, and 2.52, respectively; the MRFPEs (%) are 1.938, 5.83, and 8.06, respectively, and the CMRPEs (%) are 2.128, 2.956, and 3.905. Similar to the previous example, although the FANGBM yields the lowest simulation error, while the NFOGBM produces the lowest prediction error and combined error, and the corresponding modeling curve fits the original data the best; therefore, among these three models, the NFOGBM provides the best modeling accuracy.

(6) The modeling results for CMD are shown in Table 7, and the fitting and error plots are shown in Figure 8. The MRPESs (%) of the NFOGBM, FANGBM, and NGBM are 4.112, 3.406, and 4.459, respectively; the MRFPEs (%) are 0.519, 4.104, and 11.146, respectively, and the CMRPEs (%) are 3.727, 4.007, and 6.688, respectively. In this example, the NGBM does not capture the changes in the new data, and the corresponding modeling curve displays an upwards trend in the prediction phase, seriously deviating from the original data. Both the NFOGBM and the FANGBM can use all available information in modeling, and the NFOGBM greatly improves upon the traditional grey Bernoulli model by utilizing new data, yielding the lowest prediction error and comprehensive error, and providing the optimal modeling accuracy.

(7) The modeling results for PSESH are shown in Table 8, and the fitting and error plots are shown in Figure 9. The MRPESs (%) of the NFOGBM, FANGBM, and NGBM are 0.64, 0.708, and 0.806, respectively; the MRFPEs (%) are 0.373, 0.485, and 3.172, respectively, and the CMRPEs (%) are 0.573, 0.652, and 1.397, respectively. For the NFOGBM and FANGBM, all three errors are less than 1%, which indicates that these two models perform well for this example. Although the NGBM yields improved simulations in this case compared to those in previous examples, its prediction accuracy is low. Moreover, the NFOGBM yields the lowest simulation, prediction, and synthesis errors, and the highest modeling accuracy, achieving a notable improvement over the traditional grey Bernoulli model.

In summary, the FANGBM improves the modeling performance of the NGBM in some cases, but overfitting problems occur in some cases, while the NFOGBM yields low errors, high prediction performance, and the best modeling accuracy in all seven examples because the new background value optimization approach can further reduce the errors in the modeling process and improve the modeling accuracy. The opposite-direction cumulative operation can further enhance the ability of the model to utilize new information, can use grey information to capture the latest change trends in the system, and can also solve the overfitting problem existing in the FANGBM. Thus, the improved grey Bernoulli model based on these two approaches can effectively use the latest data to produce both accurate prediction results that capture the trends of the new data as well as results that are highly compatible with the original data. Thus, the proposed opposite-directional cumulative operation and background value optimization method are reasonable and effective, and the new information prioritization concept can further improve modeling performance.

### 3.3. Forecasting and Analysis

**Figure 10:**
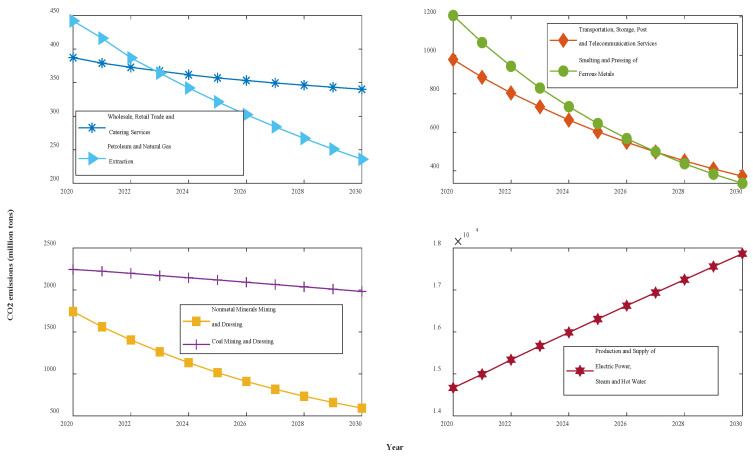
CO_2_ emissions curves for the next 11 years in Shaanxi.

According to the model results for the seven groups of data above, the NFOGBM yields the highest modeling accuracy among the models considered. Thus, the NFOGBM was applied and combined with a metabolic concept to forecast the CO_2_ emissions of the seven industries from 2020 to 2030. We first modeled and forecasted the data for 2020 using six datasets from 2014–2019, and then eliminated the old data from 2014 to add the newly predicted 2020 data. This process was then repeated for 2021–2030. The forecasting results are shown in Table 9 and Figure 10.

**Figure 11:**
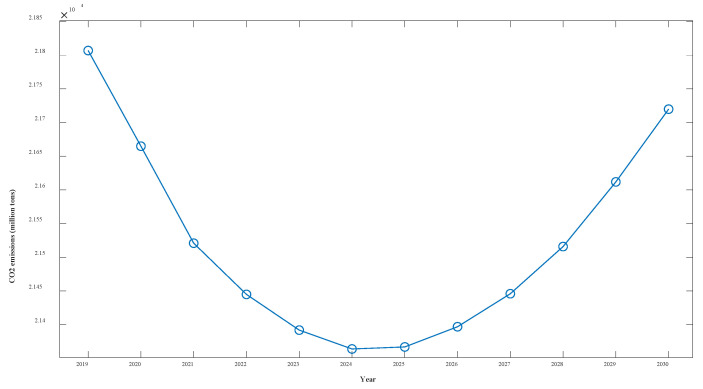
Overall CO_2_ emissions curve for Shaanxi Province over the next 11 years.

The forecasting results show that under the various grey conditions in effect, CO_2_ emissions are projected to decline in all six sectors—WRTCS, PNGE, TSPTS, SPFM, NMMD, and CMD—and to further increase in the PSESH sector. CO_2_ emissions in WRTCS are projected to decline at an average rate of 1.3% per year and are projected to decline by 55.5 million tons. CO_2_ emissions in PNGE are projected to decline at an average rate of 4.6% per year and are expected to decline by 2.39 million tons by 2030. CO_2_ emissions in TSPTS are projected to decline at an average rate of 5.9% per year and are expected to decline by 6.99 million tons by 2030. CO_2_ emissions in SPFM are projected to decline at an average rate of 6.8% per year and are expected to decline by 10.19 million tons by 2030. CO_2_ emissions in NMMD are projected to decline at an average rate of 6.3% per year and are expected to decline by 13.35 million tons by 2030. CO_2_ emissions in CMD are projected to decline at an average rate of 1.2% per year and are expected to decline by 2.92 million tons by 2030. CO_2_ emissions in PSESH are projected to increase at an average rate of 2.3% per year and are expected to rise by 25.52 million tons by 2030. For the seven industries combined, as shown in Figure 11, the total CO_2_ emissions curve shows a convex shape with little overall change, with the value expected to decline by 0.4% by 2030.

From the forecast results, four industries, PNGE, TSPTS, SPFM, and NMMD, show a larger proportional CO_2_ emissions decrease, with emissions expected to drop by more than 50% by 2030. This situation will undoubtedly contribute significantly to the accomplishment of CO_2_ emissions reduction and carbon neutrality targets, but it is not consistent with the actual situation in Shaanxi Province, where the pace of CO_2_ emissions reduction is too fast. The excessively fast reduction of CO_2_ emissions may be due to technological improvements and the further use of clean energy, but the possibility that it is caused by the reduction of energy use by industry, i.e., industry reducing emissions for the sake of reducing emissions, cannot be excluded, and this approach is contradictory to the overall goal of improving people’s economic well-being. Enterprises should steadily reduce their CO_2_ emissions by using clean energy or upgrading technology while ensuring the normal development of the industry. The government should appropriately reduce the CO_2_ emissions reduction targets for these four industries to ensure the healthy development of enterprises. The decline in CO_2_ emissions in WRTCS and CMD is more normal, indicating that the existing policies of enterprises and the government are more applicable to these sectors, and should continue to be implemented.

Considering the overall CO_2_ emissions of the seven industries, the curve shows a trend of decreasing and then increasing because the increase in CO_2_ emissions from PSESH in the later period exceeds the decrease in CO_2_ emissions from other industries. Obviously, the most serious CO_2_ emissions problem among the seven industries is found in PSESH, which contributes more than half of the total CO_2_ emissions of the seven industries and still maintains an increasing state. With the continuous improvement of the economic level and the improvement of people’s living standards, the demand for electricity and heat increases, which is inevitable. How to maintain or even reduce the CO2 emissions level while maintaining the normal supply of electricity and heat is a challenge. Enterprises should further improve the use of clean energy and technology to ensure production, and the government should further increase the control of CO_2_ emissions in the PSESH industry to enforce pressure. Notably, this paper verifies the validity of the NFOGBM(1,1,α,β) model, which can be applied to the prediction of economic and social fields except CO_2_ emissions prediction.

## 4. Conclusions

In this paper, a novel fractional-order inverse cumulative operation method and background value optimization method are proposed, and a new grey model NFOGBM(1,1) is combined with the fractional-order nonlinear Bernoulli model FANGBM(1,1). Then, the validity of the NFOGBM(1,1,α,β) model is verified using carbon emission data from seven major industries in Shaanxi Province, China. Next, using NFOGBM(1,1,α,β) and a metabolic concept, the CO_2_ emissions of the seven industries from 2020 to 2030 are predicted. The following conclusions are obtained. First, the new background value optimization approach is effective and reasonable, and it can improve the performance of the traditional model to achieve accurate prediction. Second, the combination of the new opposite-direction cumulative optimization approach with the traditional grey Bernoulli model is effective, and this approach can utilize new information and solve the overfitting problem of the FANGBM(1,1) model. Third, the NGBM(1,1) model does not sufficiently utilize new information, and the FANGBM(1,1) model produces overfitting in some cases. The NFOGBM(1,1,α,β) model provides better predictions than and outperforms the NGBM(1,1) and FANGBM(1,1) models, notably improving the prediction accuracy of the traditional grey model. Fourth, the prediction results show that under the current conditions, in 2020–2030, the CO_2_ emissions from the production and supply of electricity and heat will further increase and are expected to reach 17,865,000 tons by 2030. The CO_2_ emissions of the remaining six examined industries will all decrease. Therefore, to successfully achieve the carbon peak target in Shaanxi Province, the primary problem that needs to be solved is the excessive and rapid growth of CO_2_ emissions caused by the production and supply of electricity and heat.

Various types of grey models exist, and in this paper, only the new opposite-directional cumulative and background value optimization approach is applied to the traditional grey Bernoulli model. The effect of combining these two optimization methods with other models is not yet known. Moreover, only the CO_2_ emissions of each industry in Shaanxi Province are predicted, and the corresponding emission problems are discussed; however, solutions to these problems must be further explored in future research.

## Figures and Tables

**Table 1:** CO_2_ emissions from 7 industries in Shaanxi, 2011–2019 (million tons).

Industries	2011	2012	2013	2014	2015	2016	2017	2018	2019
WRTCS	450	450	430	440	430	415	414	403	395
PNGE	650	600	620	590	574	622	511	506	474
TSPTS	1820	1870	1440	1510	1494	1295	1299	1168	1070
SPFM	340	2090	2140	2250	2030	1773	1708	1515	1354
NMMD	3790	2510	2790	2980	2762	2363	2379	2119	1927
CMD	1080	1240	1610	1710	2389	2412	2322	2307	2274
PSESH	9870	11,820	12,770	13,210	12,896	13,307	13,594	13,964	14,313

**Table 2:** Modeling results for WRTCS.

Year	Raw Data	NFOGBM	FANGBM	NGBM
		r = 0.7408 η = −0.2165 α = 0.7907 β = 0.3508	r = 1.1061 η = 0.1782	η = 0.1075
2012	450	450.000	450.000	450.000
2013	430	430.000	430.000	431.269
2014	440	433.610	434.823	434.782
2015	430	430.000	430.000	430.002
2016	415	422.937	421.099	421.238
2017	414	413.996	410.047	410.291
MRSPE (%)		0.561	0.600	0.647
2018	403	403.972	397.784	398.099
2019	395	393.318	384.847	385.211
MRFPE (%)		0.333	1.932	1.847
CMRPE (%)		0.504	0.933	0.947

**Table 3:** Modeling results for PNGE.

Year	Raw Data	NFOGBM	FANGBM	NGBM
		r = 2.0809 η = 0.5081 α = 0 β = 0.4364	r = 0.4235 η = −1.7308	η = 0.0062
2012	600	600.000	600.000	600.000
2013	620	620.000	620.000	620.023
2014	590	602.965	594.788	601.671
2015	574	573.998	574.000	583.159
2016	622	542.429	556.309	564.901
2017	511	511.002	540.707	547.035
MRSPE (%)		2.498	2.864	3.302
2018	506	480.694	526.614	529.621
2019	474	451.865	513.678	512.683
MRFPE (%)		4.836	6.222	6.415
CMRPE (%)		3.083	3.704	4.080

**Table 4:** Modeling results for TSPTS.

Year	Raw Data	NFOGBM	FANGBM	NGBM
		r = 0.5078 η = −0.3483 α = 1 β = 0.1420	r = 0.5782 η = 0.3862	η = 0.4281
2012	1870	1870.000	1870.000	1870.000
2013	1440	1440.000	1440.000	1440.000
2014	1510	1510.442	1484.607	1494.571
2015	1494	1474.986	1428.741	1457.663
2016	1295	1396.244	1361.569	1370.945
2017	1299	1299.000	1291.869	1259.307
MRSPE (%)		1.520	1.957	2.062
2018	1168	1195.227	1222.270	1137.943
2019	1070	1091.290	1153.913	1016.081
MRFPE (%)		2.160	6.244	3.806
CMRPE (%)		1.680	3.028	2.498

**Table 5:** Modeling results for SPFM.

Year	Raw Data	NFOGBM	FANGBM	NGBM
		r = 0.5185 η = −0.3568 α = 0.6121 β = 0.3518	r = 0.3259 η = 5	η = 0.2786
2012	2090	2090.000	2090.000	2090.000
2013	2140	2140.000	2140.000	2174.925
2014	2250	2142.603	2199.429	2161.105
2015	2030	2030.000	2023.098	2030.006
2016	1773	1875.543	1820.854	1853.529
2017	1708	1708.000	1667.893	1663.842
MRSPE (%)		1.759	1.272	2.118
2018	1515	1540.811	1555.693	1476.867
2019	1354	1380.688	1469.781	1300.579
MRFPE (%)		1.837	5.619	3.231
CMRPE (%)		1.779	2.359	2.397

**Table 6:** Modeling results for NMMD.

Year	Raw Data	NFOGBM	FANGBM	NGBM
		r = 0.4399 η = −0.4782 α = 0.8727 β = 0.2841	r = 0.2703 η = 5	η = 0.3781
2012	2510	2510.000	2510.000	2510.000
2013	2790	2790.000	2790.000	2789.999
2014	2980	2867.592	2913.722	2887.712
2015	2762	2762.000	2708.019	2759.928
2016	2363	2584.477	2482.463	2531.133
2017	2379	2379.000	2313.422	2263.988
MRSPE (%)		2.191	1.998	2.520
2018	2119	2166.631	2187.547	1991.777
2019	1927	1958.364	2089.350	1732.048
MRFPE (%)		1.938	5.830	8.060
CMRPE (%)		2.128	2.956	3.905

**Table 7:** Modeling results for CMD.

Year	Raw Data	NFOGBM	FANGBM	NGBM
		r = 1.5927 η = 0.7403 α = 0.3994 β = 0.4578	r = 0.2027 η = 5	η = 0.5327
2011	1080	1080.000	1080.000	1080.000
2012	1240	1239.999	1240.000	1239.999
2013	1610	1610.785	1565.984	1612.144
2014	1710	1915.161	1956.694	1911.395
2015	2389	2133.624	2271.554	2144.367
2016	2412	2266.142	2379.466	2318.885
2017	2322	2322.002	2331.755	2442.800
MRSPE (%)		4.112	3.406	4.459
2018	2307	2314.369	2240.143	2523.507
2019	2274	2257.191	2153.466	2567.747
MRFPE (%)		0.519	4.104	11.146
CMRPE (%)		3.727	4.007	6.688

**Table 8:** Modeling results for PSESH.

Year	Raw Data	NFOGBM	FANGBM	NGBM
		r = 1.1448 η = 0.0766 α = 0.3270 β = 0.7671	r = 0.3870 η = −2	η = 0.0199
2012	11,820	11,820.000	11,820.000	11,820.000
2013	12,770	12,770.000	12,824.334	12,770.097
2014	13,210	12,871.212	12,925.510	12,997.436
2015	12,896	13,060.541	13,095.675	13,177.899
2016	13,307	13,307.000	13,323.063	13,336.724
2017	13,594	13,594.000	13,594.000	13,483.178
MRSPE (%)		0.640	0.708	0.806
2018	13,964	13,912.671	13,900.624	13,621.740
2019	14,313	14,257.941	14,238.462	13,754.926
MRFPE (%)		0.373	0.485	3.172
CMRPE (%)		0.573	0.652	1.397

**Table 9:** Forecasted CO_2_ emissions of 7 industries in Shaanxi (million tons).

Year	WRTCS	PNGE	TSPTS	SPFM	NMMD	CMD	PSESH
2020	387.22	442.01	978.06	1206.63	1741.61	2244.03	14,665.29
2021	379.02	416.18	885.34	1066.04	1560.50	2223.37	14,990.31
2022	372.87	386.93	804.16	942.82	1403.92	2197.96	15,336.01
2023	367.23	364.27	732.10	829.72	1263.03	2170.95	15,664.90
2024	361.76	342.07	663.44	732.84	1133.18	2144.73	15985.98
2025	357.00	321.44	602.83	645.38	1014.53	2119.37	16,306.14
2026	353.17	302.32	547.97	567.24	908.97	2091.63	16,626.06
2027	349.45	284.19	497.23	498.27	816.42	2064.35	16,936.58
2028	346.24	266.95	450.98	436.58	732.78	2036.93	17,245.18
2029	343.14	250.98	409.52	382.36	658.42	2009.77	17,557.40
2030	340.15	235.89	371.48	334.65	591.89	1981.65	17,864.54

## Data Availability

The datasets of this paper are available from the corresponding author on reasonable request.
